# Effectiveness of Flapless Static Computer-Aided Implant Surgery in Post-Oncological Patients: A Retrospective Study

**DOI:** 10.3390/jcm15197691

**Published:** 2026-10-04

**Authors:** Edoardo Brauner, Andrea Albini, Giulia Amelina, Federico Laudoni, Eleonora Sforza, Nicola Pranno, Francesca De Angelis, Stefano Di Carlo

**Affiliations:** 1 Department of Oral and Maxillofacial Sciences, Sapienza University of Rome, Via Caserta 6, 00161 Rome, Italy; edoardo.brauner@uniroma1.it (E.B.); albini.1940449@studenti.uniroma1.it (A.A.); federico.laudoni@gmail.com (F.L.); sforza.1898459@studenti.uniroma1.it (E.S.); nicola.pranno@uniroma1.it (N.P.); francesca.deangelis@uniroma1.it (F.D.A.); stefano.dicarlo@uniroma1.it (S.D.C.); 2 Implanto-Prosthetic Unit, Policlinico Umberto I, Viale Regina Elena I 287b, 00161 Rome, Italy

**Keywords:** flapless, implant surgery, S-CAIS (static computer-aided implant surgery)

## Abstract

**Objectives:** The present retrospective study evaluates the clinical outcomes of a standardized implant-supported rehabilitative workflow based on flapless static computer-assisted implant surgery (S-CAIS) in post-oncological patients, compared with a non-oncological cohort treated using the same approach. **Methods:** In total, 26 patients were included in this study. They were divided into oncological patients (11) and non-oncological patients (15). The protocol used for digital implantoprosthetic surgical planning was the same in both groups, and it involved different decision-making algorithms depending on whether the patient was partially or completely edentulous. Only flapless surgery was performed in this study. In the case of intercalated edentulism without loss of vertical dimension, implant-prosthetic rehabilitation proceeded directly; by contrast, in the case of severe occlusal and vertical dimension alterations, temporary rehabilitation was performed to stabilize occlusion. For totally edentulous patients, the absence of stable landmarks complicated the alignment of the CT dental scan/CT cone beam, so the Evobite device was used. Implant survival was evaluated over a follow-up ranging from 1 to 3 years. The primary outcome was implant survival, defined as the proportion of functioning implants among the total number of implants placed in each group. **Results:** The implant survival rate was 96.2% in oncological patients and 98.8% in non-oncological patients. **Conclusions:** High implant survival rates were observed in post-oncological patients treated with flapless S-CAIS, although these findings should be interpreted within the limitations of the retrospective study design and selected patient population.

## 1. Introduction

Oral and pharyngeal malignancies are the sixth most common cancers worldwide and, in 2018, accounted for approximately 3.6% of all new cancer cases [1,2,3]. Patients with head and neck cancer are generally treated with ablative surgery, radiotherapy, chemotherapy, or a combination of these. Surgical treatment of malignancies involving the oral cavity often results in an altered anatomical situation, which may severely hinder normal stomatognathic functions [4]. Rehabilitation of these patients is challenging, so it is important to define the most suitable prosthetic solution [4,5]. Conventional prostheses may not represent the optimal treatment option because of the altered oral anatomy, extensive tissue loss following ablative surgery, and the adverse effects of adjuvant cancer therapy, such as mucositis, xerostomia, and alterations of the bone healing processes [6,7]. Therefore, dental implants may represent a more effective solution for oral rehabilitation. However, implant failure has been reported more frequently in post-oncological patients if the right precautions are not taken [8,9,10]. Static computer-assisted implant surgery (S-CAIS) enables virtual implant planning based on three-dimensional CT or CBCT imaging. This approach allows accurate implant positioning according to the available bone volume, anatomical structures, and prosthetic requirements, thereby facilitating prosthetically driven implant planning [11,12,13,14,15]. A key principle of S-CAIS is minimally invasive surgery. While minimal invasiveness in implantology may encompass several aspects, in implant placement it mainly refers to flapless procedures. Indeed, flapless implant placement has been shown to reduce surgical trauma and operative time, thereby resulting in less patient discomfort and postoperative morbidity [16,17,18]. In patients who have undergone surgical treatment for head and neck cancer, residual bone availability may be substantially compromised [19]. Therefore, in patients with sufficient residual bone volume to allow implant placement, S-CAIS may facilitate the prosthetically driven use of the available bone while minimizing surgical trauma. The present retrospective study evaluates a standardized implant-supported rehabilitation workflow adopted in our clinical practice for post-oncological patients. The primary aim was to evaluate the clinical outcomes observed in post-oncological patients treated with this workflow. A secondary aim was to compare the observed implant survival and clinical outcomes with those of a non-oncological cohort treated using the same workflow.

## 2. Materials and Methods

This retrospective study was conducted at the Implant Prosthodontics Unit, Department of Head and Neck Surgery, Sapienza University of Rome, Umberto I University Hospital, Rome, Italy. Patients treated at our unit between 2020 and 2025 were retrospectively identified from the clinical database and selected according to the eligibility criteria. A total of 26 patients were included in this study according to the following inclusion criteria: patients with partial or complete edentulism, adequate residual bone volume (with no need for bone grafting or regenerative procedures), non-smokers, good oral hygiene, and compliance with scheduled follow-up visits. Only patients with documented regular follow-up and at least 12 months of clinical monitoring were included, as adequate compliance with scheduled recall visits and maintenance of good oral hygiene and health status were considered essential for reliable outcome assessment. No patients included in the final study cohort were lost to follow-up. The exclusion criteria were patients with systemic conditions contraindicating surgical intervention, completion of chemotherapy and/or radiotherapy less than 12 months before implant surgery, and incomplete or insufficient documentation. The 12-month interval was adopted as a prudential criterion in our clinical practice, as no consensus exists in the literature regarding the optimal timing for implant placement after radiotherapy. Treatment planning was based on a comprehensive assessment of individual oncological and local risk factors, including previous radiotherapy, residual bone, and soft-tissue conditions. In irradiated bone, these considerations are particularly important, as the radiation dose and the resulting alterations in bone vascularity and soft-tissue healing capacity may influence the choice of implant site, timing, and surgical approach. Accordingly, for all irradiated patients, the radiation dose and the anatomical distribution of the irradiated field were carefully assessed during preoperative planning to guide treatment decisions on an individual basis [8,9,10,11]. The enrolled patients were divided into two groups: 11 oncological patients (Table 1) and 15 non-oncological patients (Table 2). The oncological group consisted of patients with a documented history of malignancy involving the oral cavity, all of whom had completed the appropriate oncological treatment. Patients in the oncological group underwent different oncological treatments. Specifically, one patient underwent radical neck dissection, five received chemotherapy, four underwent radiotherapy, and five underwent reconstructive surgery (Table 3). In total, 15 patients were partially edentulous, and the remaining patients were completely edentulous. The non-oncological cohort consisted of patients treated at the same Implant Prosthodontics Unit during the study period who met the eligibility criteria for implant-supported rehabilitation and were retrospectively identified from the clinical database. The digital protocol used for implant-prosthetic surgical planning was the same for both groups, including different decision-making algorithms depending on whether the patient was partially or completely edentulous. In this study, only flapless surgery was performed.

All implants were manufactured by Zimvie. Virtual implant planning was performed using RealGUIDE software (ZimVie), and the surgical guides were manufactured by ZimVie according to the digitally planned workflow. All surgical procedures were performed by the same experienced operator in an outpatient setting under antibiotic prophylaxis. No immediate loading protocol was adopted. Follow-up included routine clinical and radiographic examinations, with visits scheduled more frequently during the early postoperative period and subsequently at regular intervals. The timing of healing-abutment placement was determined on an individual basis according to the local soft-tissue conditions and the specific clinical characteristics of each patient.

The surgical guide design was adapted to the patient’s dental status. In partially edentulous patients, when sufficient teeth were available to provide additional stabilization, the guide was supported by both teeth and mucosa. In completely edentulous patients, a fully mucosa-supported guide was used. When implants were completely submerged and local conditions allowed, the mucosal punch obtained during implant access was repositioned and sutured around the implant to preserve the soft-tissue architecture.

Given the retrospective design, the limited sample size, and the small number of implant failures, the analysis was descriptive. The primary outcome was implant survival, defined as the implant remaining functional during the observation period. Implant survival was calculated as the proportion of surviving implants among the total number of implants placed in each group. No inferential statistical tests were performed.

### 2.1. Partially Edentulous Patients

**Figure 1:**
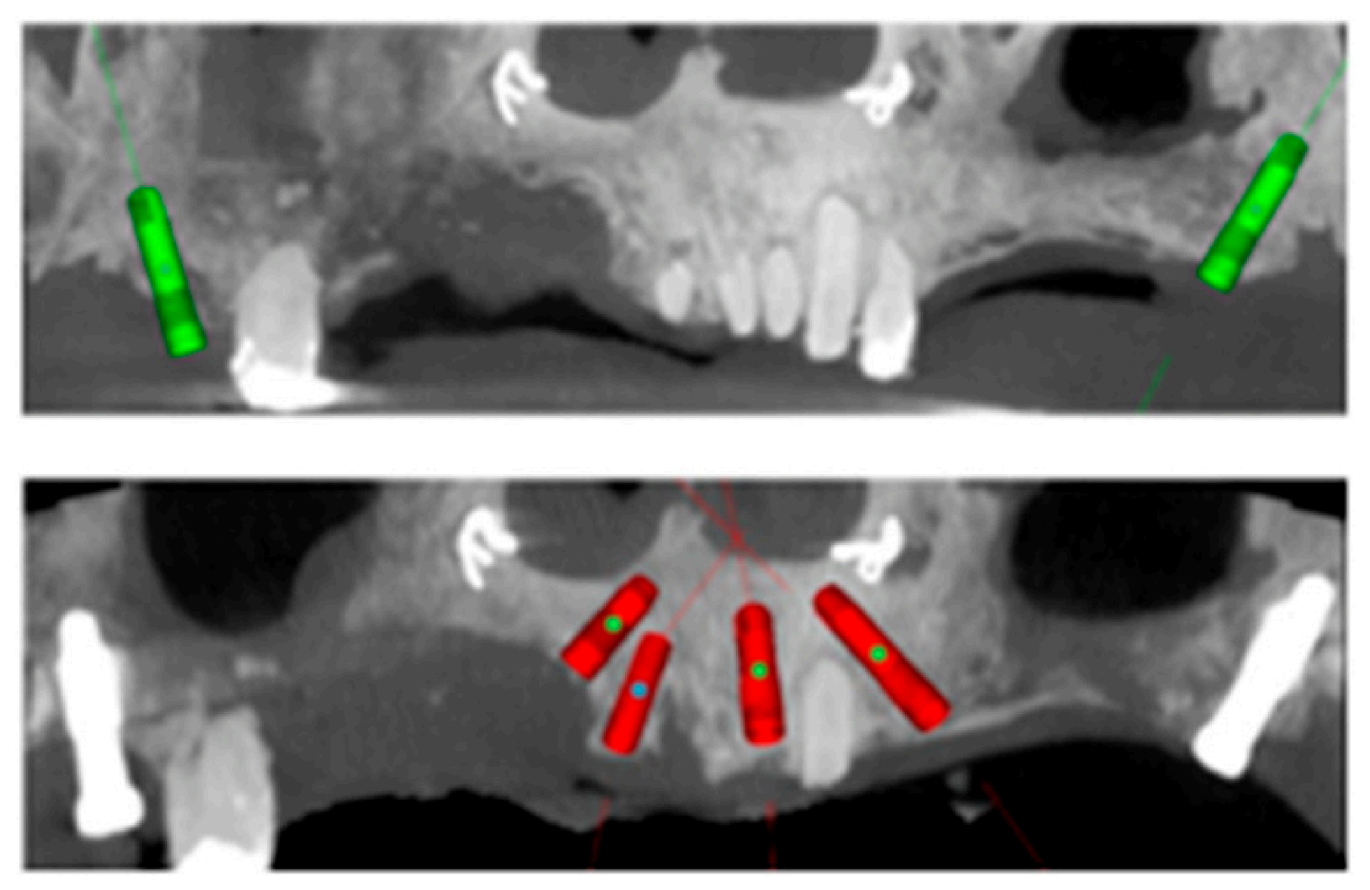
Implant planning.

**Figure 2:**
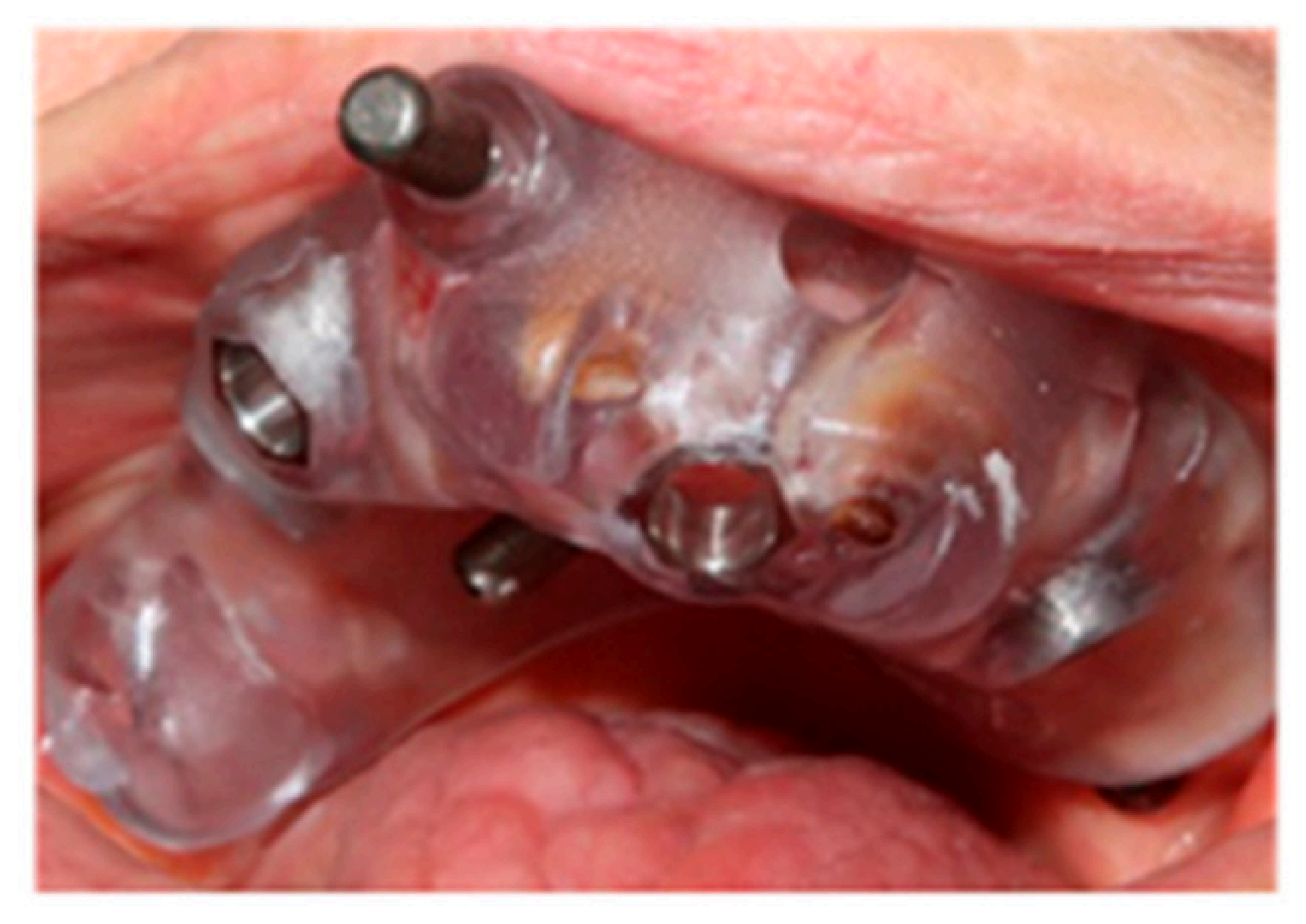
Tooth-supported surgical guide for flapless implant surgery.

**Figure 3:**
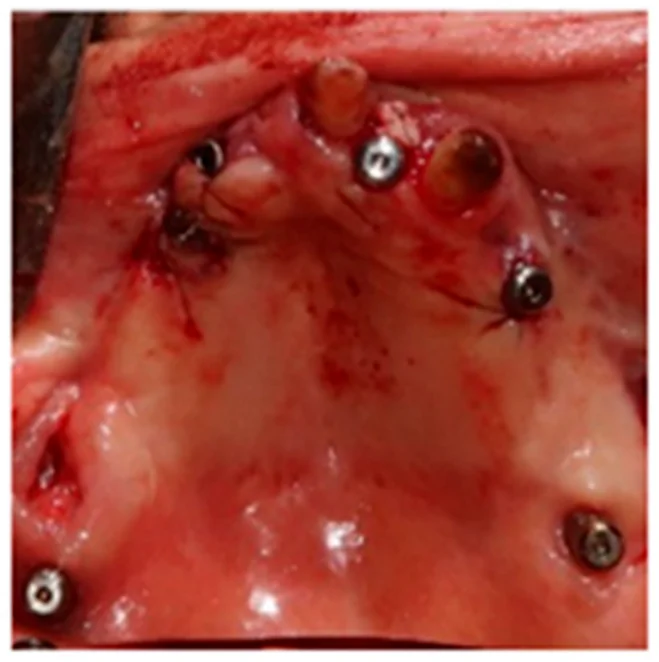
Soft tissues after implant placement.

In patients with bounded edentulous spaces and preserved occlusal relationships, implant-prosthetic rehabilitation was planned directly through a digital diagnostic wax-up. In contrast, patients presenting severe occlusal alterations or loss of vertical dimension first underwent provisional rehabilitation with a removable partial denture to restore occlusal stability before digital implant planning. The subsequent digital phase, which was the same in both subgroups, began with the acquisition of DICOM files using CBCT and scans of the dental arches in STL format and continued with their overlap on RealGUIDEZB 5.4 ZimVie software dedicated to implant surgical planning and prosthetic design. The software allows an “assisted” or “by points” overlap. In the first approach, the software autonomously matches the two files using an artificial intelligence system that recognizes common patterns occurring in both the CT scan and intraoral scans. In the second approach, the operator manually selects landmarks on the scan file and the 3D CT model; based on these landmarks, the software matches the two files: in partially edentulous patients, the presence of teeth simplifies the identification of common landmarks/patterns. Once the files had been matched, the digital wax-up or the wax-up obtained from the prosthesis scan was used for prosthetically driven implant planning, taking into account periodontal and anatomical conditions and the availability of residual bone and the position of the soft tissues (Figure 1), evaluating the availability of residual bone and the position of the soft tissues. Based on the surgical planning, the surgical guide was designed and manufactured in collaboration with the dental laboratory and adapted to the patient’s dental status. The guide was used to facilitate flapless insertion of the endosseous implants (Figure 2). The surgical approach chosen for every patient was flapless. Healing abutments or cover screws were placed according to the local soft-tissue conditions and the specific clinical characteristics of each patient (Figure 3).

### 2.2. Totally Edentulous Patients

**Figure 4:**
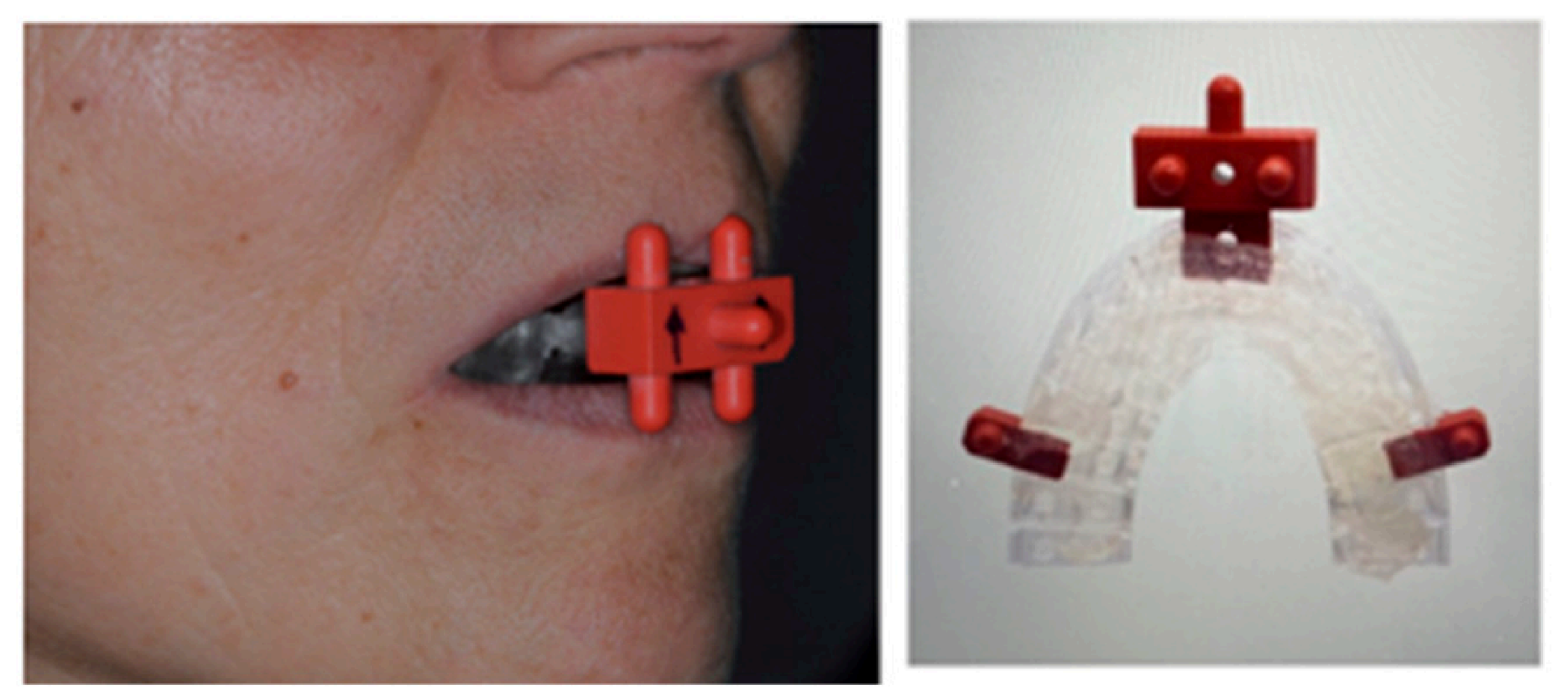
Evobite, digital registration device that facilitates the accurate alignment of CBCT and STL datasets for three-dimensional implant planning.

**Figure 5:**
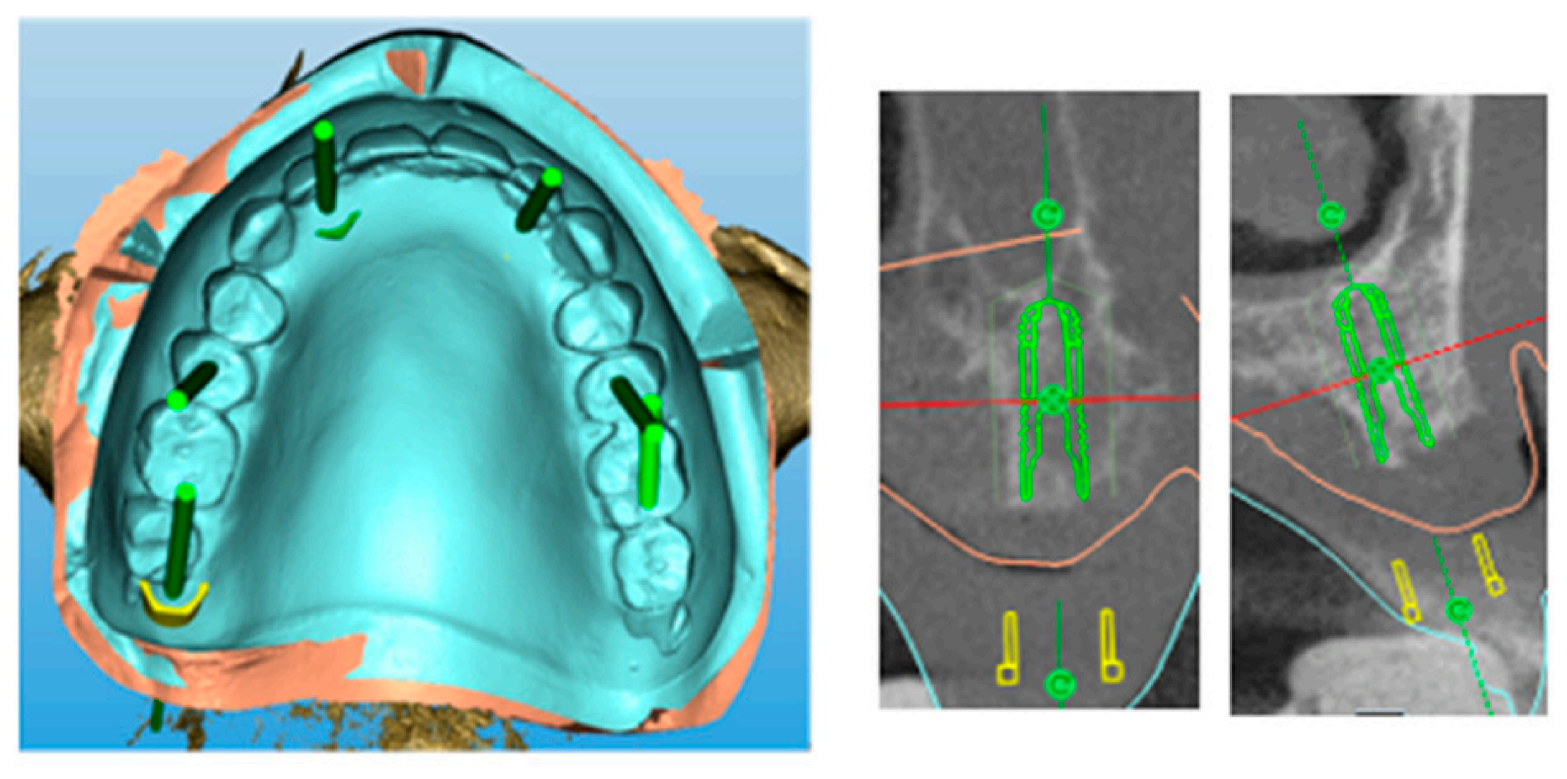
Implant planning.

**Figure 6:**
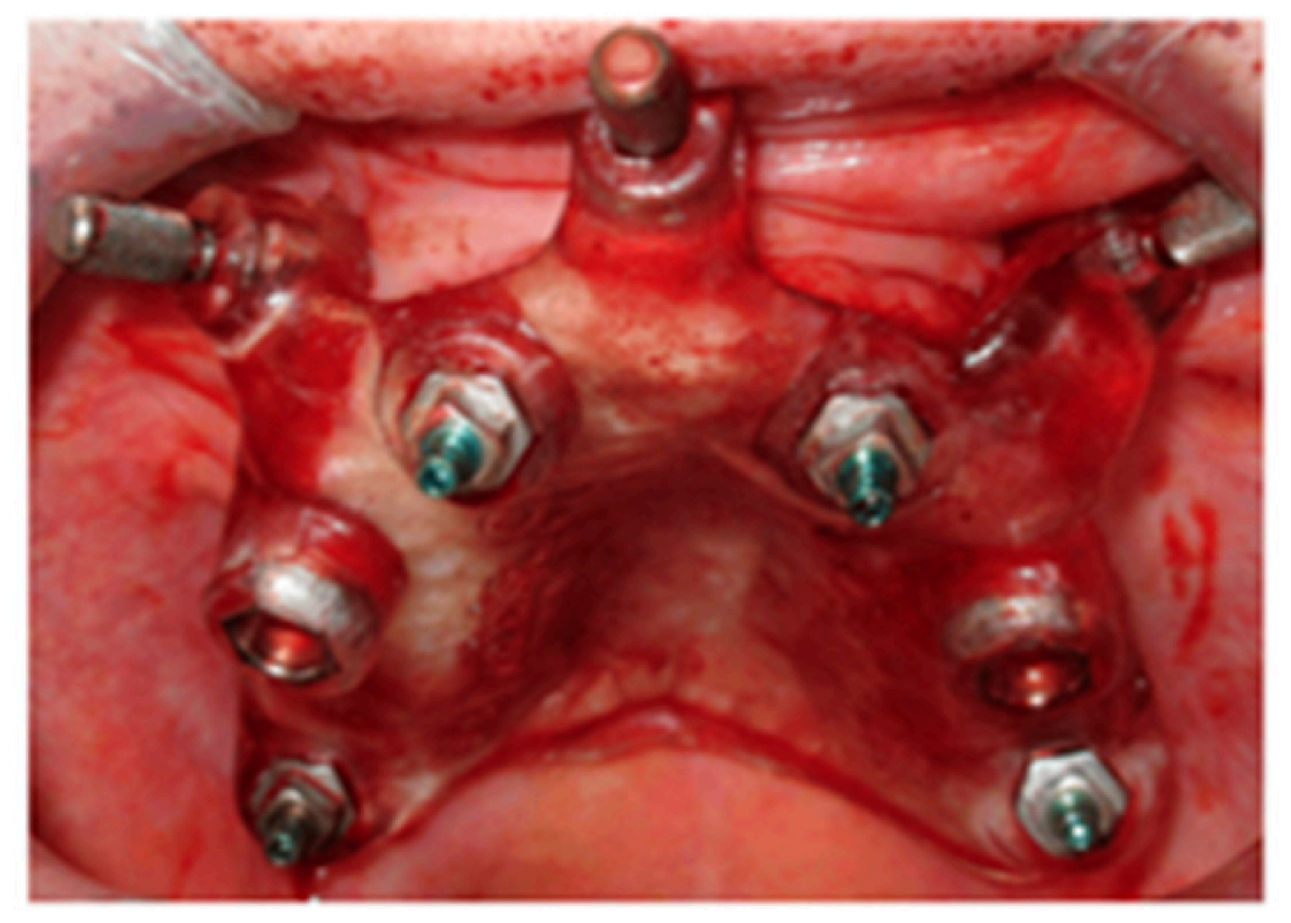
Mucosa-supported surgical guide for flapless surgery.

**Figure 7:**
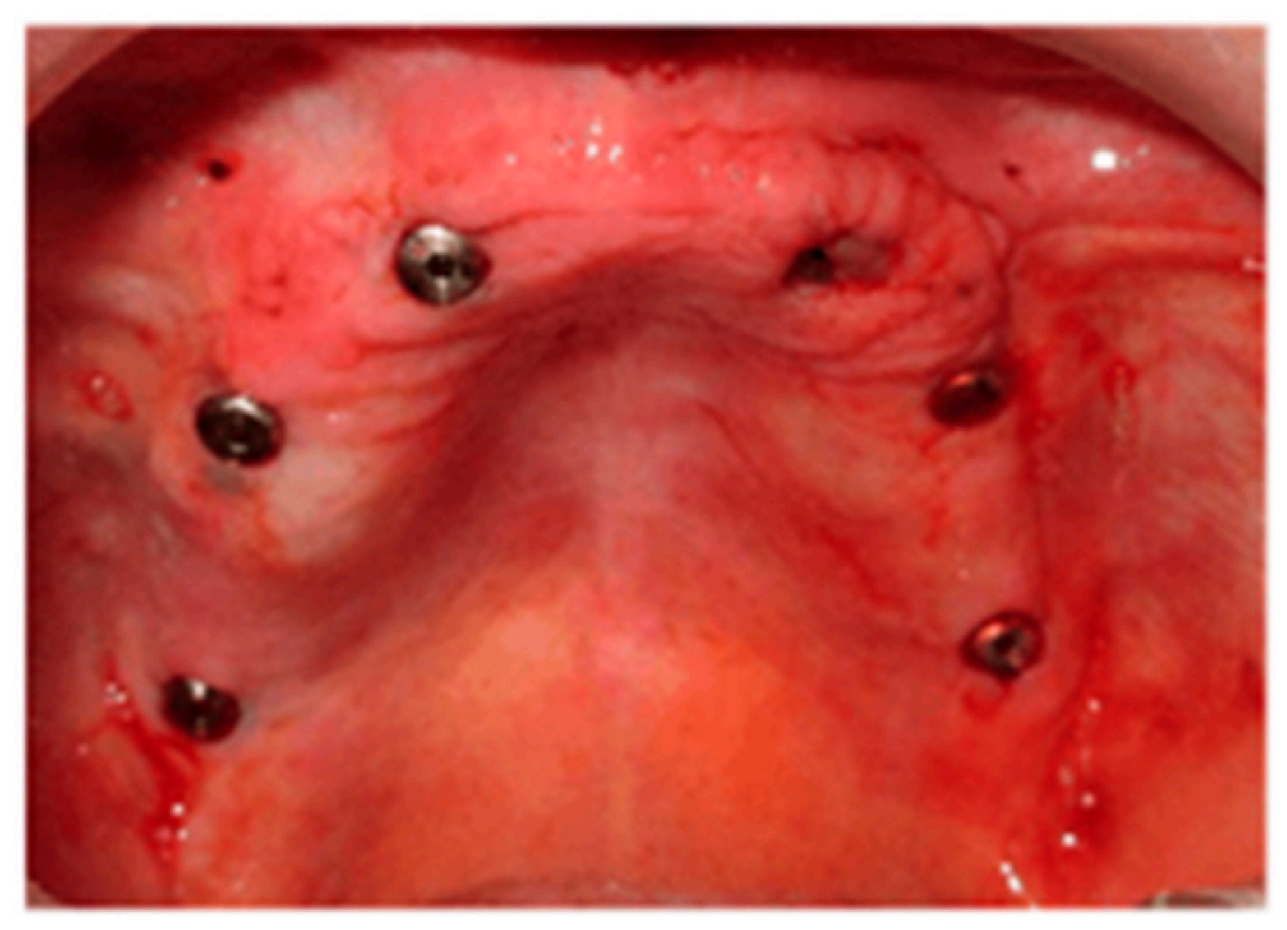
Soft tissue after implant placement.

In totally edentulous patients, rehabilitation with provisional complete dentures was performed before implant planning in order to re-establish proper occlusion, vertical dimension, esthetics, and masticatory function. Once the adaptation period was completed and the prostheses were considered clinically satisfactory, the computer-guided implant surgery workflow was initiated. Unlike partially edentulous patients, totally edentulous patients lack stable anatomical landmarks, such as the remaining teeth, which are routinely used to accurately register the DICOM dataset obtained from cone-beam computed tomography (CBCT) with the STL files generated from intraoral scanning. Moreover, intraoral scanning of completely edentulous arches is technically demanding because of the absence of fixed reference points and the mobility of the soft tissues, often resulting in scans of suboptimal accuracy. These factors may negatively affect the reliability of digital registration and, consequently, prosthetically driven implant planning. To overcome these limitations, the present protocol employed the Evobite device, consisting of an extraoral radiopaque marker connected to a fork. Using a dedicated radiolucent silicone material, the fork recorded the spatial relationship between the extraoral marker and the patient’s dental arches while the provisional complete dentures were in place. This allowed the creation of a stable and reproducible radiographic reference that could subsequently be transferred to the digital workflow. The patient underwent CBCT (or dental CT, according to the clinical indication) while wearing both the provisional complete dentures and the Evobite device (Figure 4). Particular attention was paid to the field of view (FOV), ensuring that the entire extraoral radiopaque marker was included within the scan volume, as incomplete visualization of the marker would compromise the subsequent registration procedure. At a subsequent appointment, the Evobite device was removed, and an alginate impression of the complete denture was obtained. The impression was then delivered to the dental laboratory, where the dental technician created several reference notches in the impression material. These notches served as reproducible landmarks during the fabrication of the stone casts. The impression containing the prosthesis was first poured with dental stone to obtain a cast reproducing the patient’s edentulous arches. Subsequently, after carefully removing the prosthesis from the impression without altering the reference notches, the same impression was poured again to obtain a second stone cast reproducing the complete denture. The presence of identical reference landmarks on both casts facilitated consistent correspondence between the anatomical structures and the prosthesis throughout the digital workflow. Three extraoral scans were subsequently acquired: (1) the stone cast of the complete denture together with the Evobite device, (2) the stone cast of the complete denture alone, and (3) the stone cast reproducing the edentulous arches. The resulting STL files shared identical reference landmarks, allowing for consistent superimposition of the different datasets. This workflow enabled reliable registration between the STL files and the DICOM dataset obtained from CBCT, thereby facilitating prosthetically driven virtual implant planning within the dedicated surgical planning software. Following completion of virtual planning, a tooth-supported surgical guide was not applicable because of the absence of residual teeth. Therefore, in contrast to partially edentulous patients, stabilization of the surgical guide during implant placement was achieved by means of fixation pins inserted after appropriate positioning of the guide (Figure 5, Figure 6 and Figure 7). This ensured adequate stability of the guide throughout the surgical procedure. Implant survival was evaluated during a follow-up period ranging from 1 to 3 years.

## 3. Results

**Figure 8:**
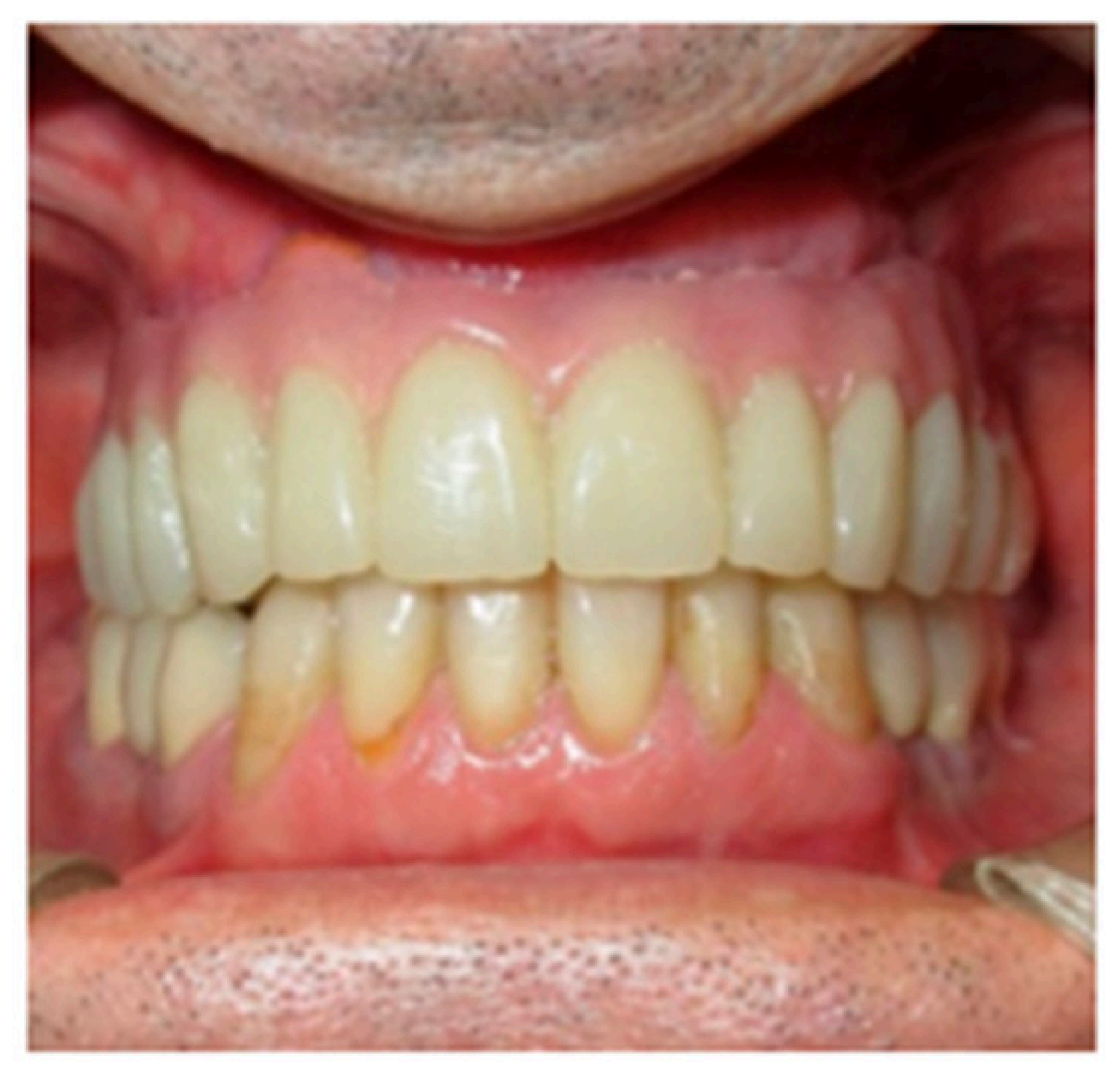
Representative clinical case of implant-supported rehabilitation in a non-oncological patient (RP4).

**Figure 9:**
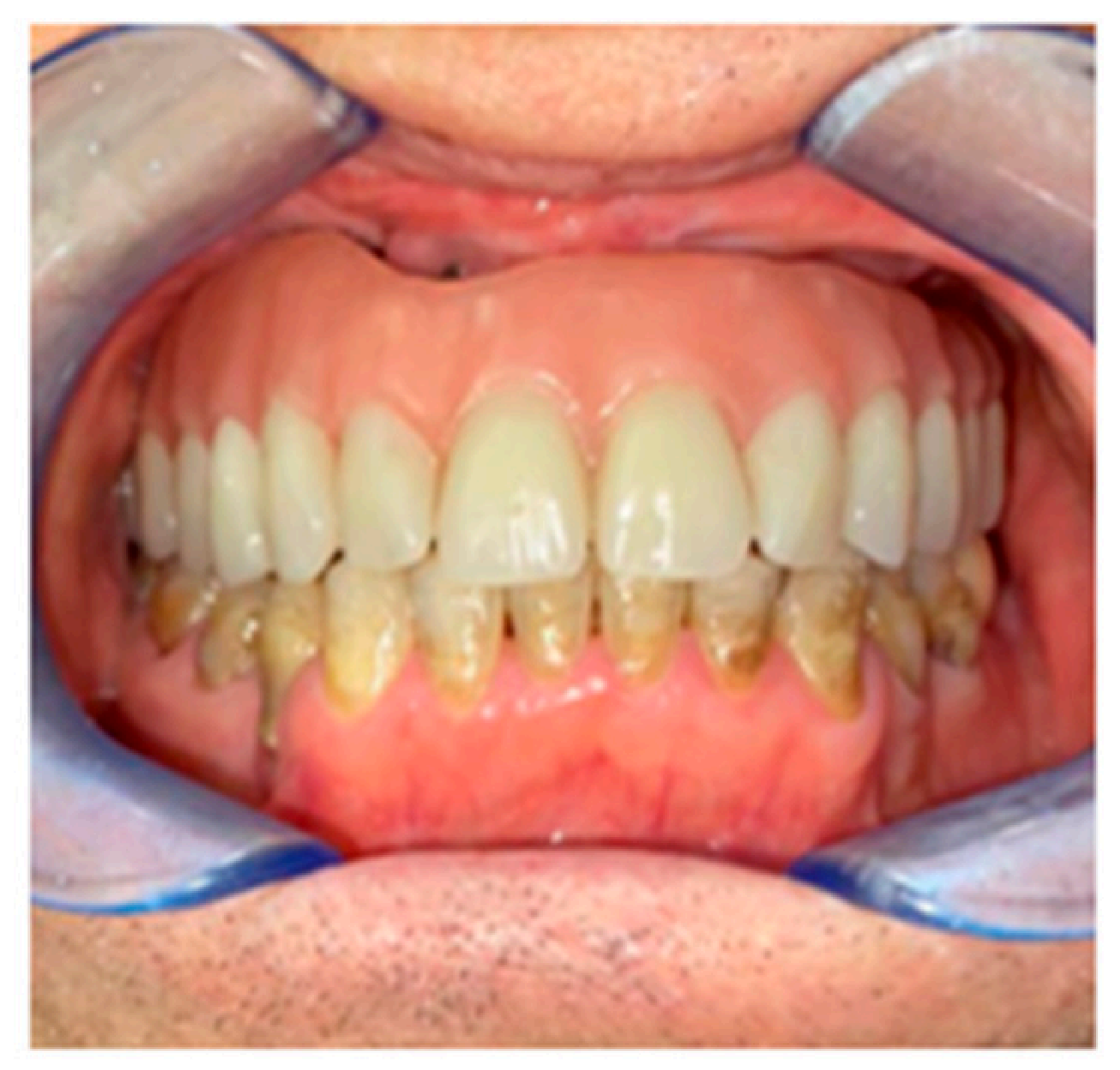
Representative clinical case of implant-supported rehabilitation in a post-oncological patient (RP4).

A total of 26 patients were included in the present study, comprising 11 oncological patients and 15 non-oncological patients. Among the 11 oncological patients, different treatment protocols had been previously performed according to the underlying disease. Specifically, one patient had undergone radical neck dissection, five had received chemotherapy, four had undergone radiotherapy, and five had undergone reconstructive surgical procedures. Regarding the preoperative clinical condition, 15 patients presented with partial edentulism, whereas 11 were completely edentulous. Overall, 133 dental implants were placed, with a reported follow-up ranging from 1 to 3 years. Of these, 52 implants were inserted in oncologic patients and 81 in non-oncologic patients. During the follow-up period, three implant failures were recorded, including two in the oncologic group and one in the non-oncologic group. Despite these three implant failures, the overall prosthetic rehabilitations remained functional, as the affected prostheses continued to be adequately supported by the remaining implants and did not require modification. Consequently, 130 implants remained functional at the end of the observation period. The implant survival rate after 1–3 years of follow-up was 96.2% in the oncologic group and 98.8% in the non-oncologic group (Table 4). Soft-tissue healing progressed uneventfully in both groups during routine clinical follow-up. Throughout the follow-up period, no local or systemic complications, including postoperative bleeding, infection, or osteonecrosis, were observed, supporting the favorable clinical outcomes observed with this approach. Figure 8 and Figure 9 show representative clinical cases of implant-supported rehabilitation performed using the same fully digital computer-guided workflow in a non-oncological and a post-oncological patient, respectively. In both cases, the patients were initially rehabilitated with a removable prosthesis, which served as the basis for the design and planning of the definitive implant-supported prosthetic restoration within the fully digital workflow.

## 4. Discussion

Post-oncological patients treated for head and neck cancer represent one of the most challenging populations for implant-supported rehabilitation because of the anatomical alterations resulting from ablative surgery and the biological effects of cancer therapies. Severe post-surgical changes frequently compromise the stability and function of removable prostheses, making implant-supported rehabilitation a valuable treatment option whenever local and systemic conditions allow. However, implant placement in these patients requires careful risk assessment [16,17,18,19,20]. Radiotherapy may impair bone vascularization and osseointegration, increasing the risk of implant failure and osteoradionecrosis. De La Plata et al. [21] reported significantly higher implant loss rates in irradiated patients compared with non-irradiated controls. In addition, radiotherapy adversely affects the peri-implant soft tissues by inducing xerostomia, mucositis, and delayed wound healing. Chemotherapy, as well as treatment with antiresorptive or antiangiogenic agents, may further compromise hard- and soft-tissue healing, thereby increasing the risk of oral complications [16,17,18,19,20,21,22,23]. In patients receiving high-dose intravenous antiresorptive or antiangiogenic therapy for bone metastases, implant placement is generally contraindicated because of the elevated risk of implant failure and medication-related osteonecrosis of the jaw, as reported by Brauner et al. [11,24] and supported by current clinical guidelines [23]. Therefore, careful patient selection and individualized risk assessment remain fundamental prerequisites for successful implant rehabilitation in this medically compromised population. Although the residual bone volume was considered adequate for implant placement without regenerative procedures, the local anatomical and biological conditions of post-oncological patients may still be altered as a consequence of previous cancer treatment. Patients requiring bone augmentation or regenerative procedures were excluded because their management would represent a different clinical treatment pathway beyond the scope of the present study. Despite these challenges, implant-supported rehabilitation remains the treatment of choice for post-oncological patients whenever local and systemic conditions permit. Appropriate patient selection, supported by validated implant failure risk assessment tools such as those proposed by Brauner et al. [24], is essential for identifying suitable candidates and optimizing clinical outcomes. The favorable results observed in the present study may be explained by several features of the adopted digital workflow. The implant survival rate observed in the present cohort was within the range of outcomes previously reported for implant rehabilitation of post-oncological patients. Prosthetically driven virtual planning may have facilitated appropriate implant positioning according to the available bone and prosthetic requirements. In addition, fully guided static computer-assisted implant surgery (S-CAIS) has been shown to achieve greater placement accuracy than freehand or partially guided approaches, with positive effects on peri-implant hard and soft tissue health. All patients in the present study were treated using the same flapless S-CAIS approach, and the present study was not designed to directly compare S-CAIS with freehand, partially guided, or open-flap surgery. Therefore, the favorable outcomes observed in our cohort should be interpreted as clinical results associated with the adopted workflow rather than as evidence of its superiority over alternative surgical approaches. Beyond implant placement, digital technologies have also expanded reconstructive possibilities in complex cases. For example, Brauner et al. demonstrated the feasibility of using CAD/CAM technology to fabricate patient-specific membranes for guided bone regeneration in severe bone defects [14], highlighting the versatility of digital workflows in implant rehabilitation. The flapless surgical approach was also used in our workflow and is generally associated with reduced surgical trauma and postoperative morbidity compared with flap elevation. In selected cases in which the implants were completely submerged and local soft-tissue conditions allowed, the mucosal punch was repositioned and sutured to preserve the peri-implant soft tissues and potentially limit soft-tissue remodeling. This strategy may be particularly useful in medically compromised patients, in whom tissue vascularization and healing capacity may already be impaired by previous cancer therapies [24,25,26,27,28,29]. In our cohort, favorable soft-tissue healing was observed following the flapless approach, in line with the biological and clinical advantages of minimally invasive surgery previously described in the literature. Moreover, flap elevation has been associated with crestal bone remodeling [30,31,32,33,34]; therefore, minimizing surgical invasiveness may contribute to preserving peri-implant bone and supporting long-term implant stability. One potential limitation of flapless surgery is the reduction of peri-implant keratinized mucosa. To address this issue, our protocol included repositioning and suturing the mucosal punch obtained during mucotomy, with the aim of preserving keratinized tissue around the implant and promoting long-term peri-implant soft-tissue stability [14,15,28]. Although the specific clinical benefits of this approach require further investigation, it represents a simple and reproducible modification that may enhance soft-tissue preservation without increasing surgical morbidity. Several limitations should be acknowledged. The retrospective design and small sample size, together with the limited number of implant failures, allow only descriptive and exploratory interpretation of the findings. The strict eligibility criteria and retrospective patient selection may also limit the generalizability of the results to the broader post-oncological population. Furthermore, the absence of a control group treated with alternative implant-placement approaches prevents direct assessment of the specific contribution of S-CAIS to the observed outcomes.

## 5. Conclusions

The present study reports high implant survival rates in post-oncological patients treated with flapless S-CAIS. Within the limitations of this retrospective study, these findings support the feasibility of this approach for implant-supported rehabilitation in appropriately selected post-oncological patients. The strict eligibility criteria and retrospective selection of patients may limit the generalizability of these findings to the broader post-oncological population, particularly to patients requiring bone augmentation or regenerative procedures or those unable to maintain regular follow-up. Given the small sample size and the absence of a control group using alternative implant-placement approaches, these findings should be considered descriptive and hypothesis-generating. Further prospective controlled studies with larger cohorts and longer follow-up are required to confirm these preliminary findings.

## Figures and Tables

**Table 1:** Implant and prosthetic characteristics of oncological patients.

Patient	Age/Sex	Histologic Diagnosis	N° Implants	Position	Implant Size	Prosthesis (Mish)
1.	72/M	SCC	3	2.5 2.7 4.7 †	4.1 × 8 4.1 × 8 4.1 × 8	FP1
2.	71/M	SCC	4	3.1 3.5 3.6 4.4	3.7 × 13 3.7 × 11.5 3.7 × 11.5 3.7 × 16	FP3
3.	55/F	LCC	6	1.2 1.5 1.7 2.2 2.5 2.7	3.7 × 13 3.7 × 8 3.7 × 10 3.7 × 13 3.7 × 10 3.7 × 11.5	FP3
4.	77/M	SCC	6	1.8 1.5 1.3 2.3 2.5 2.8	3.7 × 13 3.1 × 8 3.1 × 8 3.1 × 10 3.7 × 13 3.7 × 13	FP3
5.	69/M	SCC	6	1.8 1.4 1.2 2.2 2.4 2.7	3.7 × 13 3.7 × 10 3.7 × 13 3.7 × 10 3.7 × 13 3.7 × 16	FP3
6.	39/F	SCC	4	3.2 3.4 3.6 3.7	4.1 × 13 4.1 × 13 4.1 × 11.5 4.1 × 11.5	FP3
7.	71/M	SCC	5	4.7 4.6 4.4 3.4 3.6	3.7 × 10 3.7 × 11.5 3.7 × 11.5 3.7 × 11.5 3.7 × 11.5	RP4
8.	66/F	SCC	7	1.1 1.3 1.8 2.3 4.5 4.6	3.7 × 11.5 3.7 × 13 3.7 × 13 3.7 × 11.5 3.7 × 11.5 3.7 × 10	RP4
9.	45/F	SCC	2	2.1 2.7	3.7 × 10 3.7 × 13	FP3
10.	46/F	SCC	4	3.1 4.3 4.5 4.6 †	3.7 × 13 3.7 × 11.5 3.7 × 11.5 3.7 × 11.5	FP3
11.	59/M	SCC	6	1.8 2.8 1.2 1.4 2.2 2.5	3.7 × 16 3.7 × 16 3.1 × 11.5 3.7 × 10 3.7 × 13 3.7 × 16	RP4

SCC = squamous cell carcinoma; Only one implant failed during follow-up (Patient 1, implant in position 4.7). FP1: fixed prosthesis replacing only the clinical crowns; FP3: fixed prosthesis replacing the clinical crowns and missing gingival tissues. †: Implant failure during follow-up.

**Table 2:** Implant and prosthetic characteristics of non-oncological patients.

Patient	Age/Sex	N° Implants	Position	Implant Size (mm)	Prosthesis (Mish)
1.	58/F	4	1.6 1.7 2.6 2.7	3.7 × 10 3.7 × 8 3.7 × 13 3.7 × 13	FP2
2.	62/M	6	3.3 3.5 3.7 4.3 4.5 4.7	3.7 × 10 3.7 × 13 3.7 × 11.5 3.7 × 13 3.7 × 13 3.7 × 10	RP5
3.	74/M	6	3.4 3.6 3.7 4.4 4.6 4.7	3.7 × 11.5 3.7 × 10 3.7 × 10 3.7 × 11.5 3.7 × 8 3.7 × 8	FP1
4.	58/M	5	2.6 2.7 2.8 4.7 4.6	3.7 × 11.5 3.7 × 10 3.7 × 16 3.7 × 10 3.7 × 11.5	FP2
5.	67/M	6	1.8 1.5 1.2 2.2 2.5 2.8	3.7 × 13 3.7 × 10 3.7 × 13 3.7 × 11.5 3.7 × 10 3.7 × 11.5	FP3
6.	65/M	14	4.7 4.5 4.2 3.2 3.5 3.7 1.7 2.7 † 1.5 2.5 1.4 1.2 2.2 2.4	3.7 × 8 3.7 × 10 3.7 × 11.5 3.7 × 11.5 3.7 × 10 3.7 × 8 3.7 × 8 3.7 × 8 3.7 × 10 3.7 × 10 3.7 × 11.5 3.7 × 11.5 3.7 × 11.5 3.7 × 11.5	RP4
7.	63/M	6	1.2 1.4 1.7 2.2 2.4 2.7	3.7 × 13 3.7 × 13 3.7 × 10 3.7 × 11.5 3.7 × 13 3.7 × 16	FP3
8.	40/M	2	3.2 3.4	3.7 × 13 3.7 × 13	FP1
9.	72/M	4	2.3 2.4 2.5 2.6	3.7 × 13 3.7 × 11.5 3.7 × 11.5 4.1 × 11.5	FP1
10.	61/F	4	1.6 1.7 2.6 2.7	3.7 × 11.5 3.7 × 8 3.7 × 10 3.7 × 16	FP1
11.	60/F	4	2.5 2.7 4.4 4.5	3.7 × 11.5 3.7 × 8 3.1 × 13 3.1 × 13	FP1
12.	68/M	6	1.2 1.3 1.6 1.8 2.2 2.8	3.7 × 16 3.7 × 13 3.7 × 16 3.7 × 16 3.7 × 16 3.7 × 16	FP2
13.	66/F	4	1.8 2.2 2.5 2.8	3.7 × 16 3.7 × 13 3.7 × 13 3.7 × 16	FP1
14.	77/F	6	1.4 1.8 1.7 2.3 2.4 2.7	3.7 × 10 3.7 × 8 3.7 × 10 3.7 × 13 3.7 × 11.5 4.7 × 10	FP2
15.	61/F	4	1.3 1.4 1.6 1.7	3.7 × 11.5 3.7 × 11.5 3.7 × 10 3.7 × 10	FP1

FP2: fixed prosthesis replacing the clinical crowns and a portion of the root; †: Implant failure during follow-up.

**Table 3:** Cancer treatments.

	Lateral Radical Neck	Radiotherapy	Chemotherapy	Reconstruction
N° of patients	1	4	5	5
%	9.0%	36.4%	45.5%	45.5%

**Table 4:** Implant survival rates in the two patient groups.

Patient Group	Total N° of Implants	N° of Failed Implants	Implant Survival Rate
Post-oncological patients	52	2	96.2 ± 2.7%
Non-oncological patients	81	1	98.8 ± 1.2%

## Data Availability

The original contributions presented in this study are included in the article. Further inquiries can be directed to the corresponding author.
